# Free Dietary Choice and Free-Range Rearing Improve the Product Quality, Gait Score, and Microbial Richness of Chickens

**DOI:** 10.3390/ani8060084

**Published:** 2018-06-01

**Authors:** Siyu Chen, Hai Xiang, Xu Zhu, Hui Zhang, Dan Wang, Huagui Liu, Jikun Wang, Tao Yin, Langqing Liu, Minghua Kong, Jian Zhang, Shin-ichiro Ogura, Xingbo Zhao

**Affiliations:** 1 National Engineering Laboratory for Animal Breeding, Key Laboratory of Animal Genetics, Breeding and Reproduction, Ministry of Agriculture, College of Animal Science and Technology, China Agricultural University, Beijing 100193, China; jihebuluo2015@163.com (S.C.); vamyluo@126.com (H.X.); 1104010320@cau.edu.cn (X.Z.); emc42021@163.com (H.Z.); wang_d@cau.edu.cn (D.W.); xdzwjk@163.com (J.W.); gtr019@hotmail.com (T.Y.); langqing.liu@wur.nl (L.L.); kmh928335059@163.com (M.K.); 2 Laboratory of Land Ecology, Field Science Center, Graduate School of Agricultural Science, Tohoku University, Miyagi 9896711, Japan; 3 School of Life Science and Engineering, Foshan University, Foshan 528225, China; 4 Institute of Genetics and Developmental Biology, Chinese Academy of Sciences, Beijing 100101, China; 5 Institute of Animal Husbandry and Veterinary Medicine, Beijing Academy of Agriculture and Forestry Sciences, Beijing 100097, China; liuhuagui66@163.com (H.L.); zjcau@126.com (J.Z.)

**Keywords:** chicken welfare, cage rearing, free dietary choice, free-range rearing, gut microbial composition

## Abstract

**Simple Summary:**

The worldwide demand for productivity and quality meat, eggs, and other animal products is increasing. More and more people are expressing concerns relating to product quality and animal welfare. Our study aimed to provide scientific knowledge regarding how welfare factors contribute to quantity and quality of chicken. We used 400 Beijing You chickens to compare welfare factors by providing free dietary choice under cage rearing, and further comparing cage rearing with the free-range rearing system. Results showed that under cage rearing, free dietary choice of mealworms and fresh grass contributed to better meat quality, gait score and foot pad dermatitis than the conventional cage feeding and rearing system. This also gave rise to higher values of blood platelets and a richer gut microbial composition. As compared to caged chickens, free-range chickens developed better meat quality, gait score, and feather conditions, as well as a richer microbial composition. Our work provides a comprehensive understanding of welfare factors under both cage and free-range systems, and also broadens knowledge of health-related gut microbial composition in chickens.

**Abstract:**

Poultry welfare has been extensively studied; however, there is a lack of rigorous scientific knowledge relating to the different aspects of welfare factors and how this may contribute to the production quantity and product quality as well as the welfare of chickens. Therefore, we conducted an integrated study to compare welfare factors in chickens by providing free dietary choice under cage rearing, and further comparing cage rearing with free-range rearing. One hundred chickens each were allocated to a cage rearing group with conventional feeding (CC), a cage rearing group with free dietary choice of mealworms (FDM), a cage rearing group with free dietary choice of mealworms and fresh grass (FDMG), and a free-range rearing system group with free dietary choice of mealworms and fresh grass (FRMG). Results showed that under cage rearing, free dietary choice contributed to better meat quality and gait score, higher values of blood platelets, and a richer gut microbial composition, but poorer egg production than CC chickens. As compared to FDMG, FRMG chickens showed better meat quality, gait score, and feather conditions, as well as a richer gut microbial composition; however, they had poorer egg production and a poorer foot pad and foot feather condition. We conclude that free dietary choice and free-range rearing systems improve the product quality, gait score, and microbial richness of chickens.

## 1. Introduction

Globally, poultry meat has become one of the most important sources of animal protein. There are three billion hens worldwide, of which 40% are in China, making it the largest rearing project in the world since 1985 [1]. In China, there are lots of native breeds of chickens for both meat and egg production, which are favored by the majority of consumers. Still, most hens for egg production are confined in battery cages. More and more European consumers are expressing concern regarding the quality of animal products and animal welfare, and therefore, demand for high-welfare products is growing [2], including in China. Poultry welfare has been extensively studied, yet there is a lack of scientific knowledge regarding how different welfare factors contribute to the production quantity and product quality as well as welfare of chicken.

The dietary variety not only conduces to maintain homeostasis but also to reduce stress levels, and allow individual animals to have freedom to express their natural behaviors [3]. Replacing maize with *Rhizopus oryzae* improved the protein, mineral, and anti-nutritional values of raw mango seeds and did not have an adverse effect on broiler chickens’ growth performance [4]. For laying hens, free feeding choice improves the laying performance of native chickens [5], and as an environmental enrichment it has been proven to promote foraging activity, thus leading to an improvement in animal welfare [6]. Hence, free feeding choice is considered to be an important factor to improve poultry welfare in cage rearing systems. Here, worms [7] and chicory, which are widely used as dietary supplements for animals [8,9], were considered dietary variables relating to the improvement of quantity and welfare in this study.

Furthermore, the free-range rearing system is known to improve poultry welfare [10,11,12]. However, the benefits of free-range on product quality and productivity and other aspects remain elusive. For example, free-range rearing has been revealed to have negative effects on slaughter weight, but positive effects on meat quality [13] and egg quality [14], while no effect has been observed on carcass traits and meat quality in chickens [15].

In recent years, greater attention has also been given to gut microbial composition, due to its association with the promotion of health and disease in hosts [16]. There is extensive evidence that microbial colonization of the gastrointestinal tract brings benefits to chickens [17,18]. In addition, it has been demonstrated that having a normal gut microbiome moderates brain function and is essential for normal physiology and behavior in mice [19,20]. Specifically, microbial composition is influenced by diet [21] and stress [20]. Thus, we hypothesized that free dietary choice under cage rearing, as well as free-range rearing, would influence gut microbial composition and benefit animal health. Given the intensive scale of cage rearing of laying hens in China, we aimed to improve welfare by providing dietary choices under cage rearing at first. Then, we aimed to obtain a better understanding of the effect of the rearing system on productivity, product quality, welfare, and health of chickens, by comparing cage rearing to free-range chickens.

## 2. Materials and Methods

### 2.1. Animals and Experimental Design

The experimental protocols were approved by the China Agricultural University Laboratory Animal Welfare and Animal Experimental Ethical Inspection (approval number: CAU20151205-5). This study was carried out at Lvdudu Ecology Farm in Shunyi District, Beijing. Four hundred Beijing You chickens, a Chinese native breed for both meat and egg production, were used immediately after hatching at the same hatchery farm. The vaccination programs were followed by industry guidelines. All chickens were reared in a brooder house. At day 78, chickens were randomly designated into one of three cage rearing systems, or one free-range rearing system.

#### 2.1.1. Cage Rearing Treatment

Two chickens were reared per cage (length, width, and height: 0.66 m, 0.37 m, 0.5 m) on the top tier of a three-floor battery cage. The lighting schedule consisted of 16 h light and 8 h darkness, with lights switched on in the morning at 08:00 h. Chickens were fed twice daily at 09:00 h and 16:00 h and had ad libitum access to water through nipple drinkers. The dietary program of the conventional cage group (CC, *n* = 100) contained 64% corn, 20% soybean meal, 8% barn, 4% premix (i.e., amino acids, vitamins, trace elements, New Hope Group, Chengdu, Sichuan, China) and 4% limestone powder (i.e., heavy calcium carbonate power, New Hope Group, China). In the free dietary choice of mealworms feeding group (FDM, *n* = 100), 0.6% mealworm replaced soybean meal (i.e., soybean meal was 19.4%). Mealworms were commercially raised and dried to be mixed into the diet; they were visible due to their size and color. In the free dietary choice of mealworms and fresh grass group (FDMG, *n* = 100), hens were additionally fed with 11.5 g of fresh matter/head fresh chicory, (*Cichorium intybus* L.) every morning, cut into <5 cm length. The grass was only available in the morning and was directly put above the diet.

#### 2.1.2. Free-Range Rearing Treatment

The free-range rearing system (FRMG, *n* = 100) was established under the same dietary program as FDMG. Chickens were kept in an indoor area (length, width, and height: 5 m, 4 m, 2.5 m) equipped with a 50 cm depth fermentation bed and a two-floor egg box including 12 laying nests (length, width, and height: 0.3 m, 0.35 m, 0.3 m). They had free access to a hard wire fenced outdoor area (5 m × 6 m) through a floor window from Day 140. The size of the outdoor area was doubled from day 140 until the end of the experiment. The surface of the outdoor area consisted of soil without plant cover, with a 2 m^2^ sand-bath area and two perches (length, width, and height: 2 m, 0.05 m, 0.3 m). The dietary program of FRMG was identical to that of FDMG. All feed was placed in two long empty pipes (length and width: 3 m × 0.2 m). Chickens had ad libitum access to the water tank (length and width: 1.5 m × 0.2 m). The lighting schedule in the house was the same as for cage-reared chickens, with the addition of natural lighting in the outdoor area.

### 2.2. Sampling

#### 2.2.1. Production Performance and Product Quality

Production performance was calculated from Day 140 of the laying period of Beijing You Chicken. Diet and residuals were recorded daily to calculate the daily feed consumption (FC). Average egg weight (EW) was calculated using: daily total egg weight/number of eggs. Accordingly, the ratio between feeding consumption and egg weight (FC/EW) was obtained. Daily egg production was calculated using: number of laying hens/number of total hens in each group. The total outcome of soft-shelled eggs and mortality in groups was recorded. After, 30 eggs were randomly selected from each group to examine the egg yolk weight and color by EMT-5200 (Robotmation, Tokyo, Japan) at the age of 182, 224, and 266 days (at 42-day intervals). Further, 10 chickens from each group were randomly slaughtered at 280 days old (the late laying period for native breed hens). Approximately 100 g samples from both the left thigh and breast were collected to examine meat quality. For drip loss, removing the fat and connective tissue attached to the surface, the samples were trimmed into 5 cm × 3 cm × 2.5 cm and weighed (W1). The meat was stored vertically in an inflatable bag at 4 °C for 48 h and weighed as W2. Drip loss (%) = [(W1 − W2)/W1] × 100%. The right thigh and 50 g samples from the breast were stored at 4 °C. After 24 h, they were placed at room temperature and weighed for 20 g samples (W3). The samples were put into a water bath (80 °C) in a plastic bag. After the temperature reached 70 °C, the meat was removed and weighed as W4. The cooking loss (%) = [(W3 − W4)/W3] × 100%. The shear force (kg/cm^2^), was evaluated on cores (1.27 cm diameter and 3 cm length) obtained from the thickest part of the cooked samples by cutting them perpendicularly to the direction of the fiber, using an Instron model 5542 (Instron, Boston, MA, USA). Meat color was categorized into degrees of light, red, and yellow. The measurements were taken on the medial surface of each right breast and then averaged by the CIELAB method using a colorimeter (Hunter, Scan XE, Reston, WA, USA).

#### 2.2.2. Gait Score, Foot Pad Dermatitis Score, and Feather Condition Score

At day 278, 45 chickens from each group were randomly selected for gait score, foot pad dermatitis score, and feather condition score. Different assessment systems were carried out during the scoring of gait, foot pad dermatitis and feather condition. Particularly, a six-grade evaluation system was used for the gait score, in which scores of 0, 1, 2, 3, 4, and 5 represented normal walking, abnormal walking, obviously lame, able to walk under strong stimulation, unable to walk, and unable to stand, respectively. For foot pad dermatitis, scores of 0, 1, 2, 3, 4 represent no injury, slight injury on <5% of the pads, a few injuries on 5–25% of the pads, moderate injuries on 25–50% of the pads, and severe injury on >50% of the pads. Feather from the body parts of the head, neck, back, wing, vent, breast, foot and abdomen were evaluated independently. Scores of 0, 1, 2, 3, and 4 represent no feather loss, slight feather loss but not bare, size of bare patch < 3 × 3 cm, and size of bare patch > 3 × 3 cm, respectively.

#### 2.2.3. Physiological Characteristics

At day 280, a total of 5 mL blood was collected from the 10 slaughtered chickens from each group and used to measure blood lymphocyte (LYM), monocyte (MON), granulocytes (GRA), hemoglobin (HGB), platelets (PLT), mean corpuscular hemoglobin (MCH), mean corpuscular hemoglobin concentration (MCHC), red blood cell count (RBC), and mean red cell volume (MCV) by Haili Fu HF-3800 (Beijing, China).

#### 2.2.4. Gut Microbial Composition

Cecum contents were collected from 10 chickens per group for gut microbiome analyses at day 280. Total genome DNA was extracted using QIAamp Fast DNA Stool Mini Kit (QIAGEN, Hilden, Germany) following the manufacture handbook. The V4 region of 16S rDNA was amplified using the 515f/806r primer set. All PCR reactions were carried out using Phusion^®^ High-Fidelity PCR Master Mix (NEB, Beverly, MA, USA). PCR products were purified using the QIAquick Gel Extraction Kit (QIAGEN, Hilden, Germany). Libraries were generated using TruSeq^®^ DNA PCR-Free Sample Preparation Kit (Illumina, San Diego, CA, USA) following manufacturer recommendations. Sequencing was conducted on Illumina HiSeq2500 platform.

Paired-end reads were merged using FLASH v1.2.7 [22]. Chimeric sequences were removed using UCHIME algorithm [23]. Quality filtering on the raw tags was performed by QIIME v1.7.0 [24]. Operational Taxonomic Units (OTUs) were assigned using Uparse v7.0.1001 [25] with a 97% similarity threshold. Taxonomy annotation was performed by comparing sequences to the Green Gene Database.

### 2.3. Statistical Analysis

The mean ± standard error (SE) was calculated for all data. We analyzed the effect of dietary program among the cage groups (CC, FDM and FDMG), then further analyzed the effect of the rearing system under the same dietary program between FDMG and FRMG groups. The data relating to production performance, product quality and physiological characteristics was checked for normality and homogeneity of variance, of which data in line with the normal distribution was analyzed by one-way analysis of variance (ANOVA) by SAS 9.2 (SAS Inst. Inc., Cary, NC, USA), otherwise by nonparametric test by SPSS 23 (IBM, Armonk, NY, USA). In free dietary choice under cage rearing groups, a Duncan post-hoc test was used to analyze the difference among groups when significance (*p* < 0.05) was detected (the same as the analysis of microbiome). The Wilcoxon test was used to analyze gait score, foot pad dermatitis score, and feather condition score. The observed species, one of the alpha diversity analyses of gut microbial diversity, was applied to this study by QIIME v1.7.0 [25]. The data were in line with the normal distribution and analyzed by ANOVA. Beta diversity was evaluated by unweighted Unifrac distances by QIIME v1.7.0 [25] and was visualized by non-metric multi-dimensional scaling (NMDS). All values with *p* < 0.05 were regarded as statistically significant.

## 3. Results

### 3.1. Free Feed Choice under Cage Rearing Systems

#### 3.1.1. Production Performance and Product Quality

Both FC and EW were significantly higher in CC and FDMG than FDM (*p* < 0.05) (Table 1). Although egg production did not differ among the three treatments, FC/EW was higher in FDMG and FDM than CC (*p* < 0.05). The mortality of FDM chickens was lowest, followed by FDMG, and then CC chickens. FDMG chickens produced the most soft-shelled eggs, while CC chickens produced the least.

Egg yolk weight was heavier in the FDMG group than FDM at Day 182 (*p* < 0.05), while at Day 224 it was heavier in group CC than in FDMG (*p* < 0.05). Egg yolk color was significantly darker in FDMG than FDM and CC groups (*p* < 0.05). In relation to meat quality, drip loss of breast muscle was significantly greater in the FDMG group than the FDM group (*p* < 0.05). Both light and yellow values in the FDMG group were lower than in CC and FDM groups, whereas red values were higher in the FDMG group than in FDM and CC groups. No other difference was observed in meat quality.

#### 3.1.2. Gait Score, Foot Pad Dermatitis Score, and Feather Condition Score

**Figure 1:**
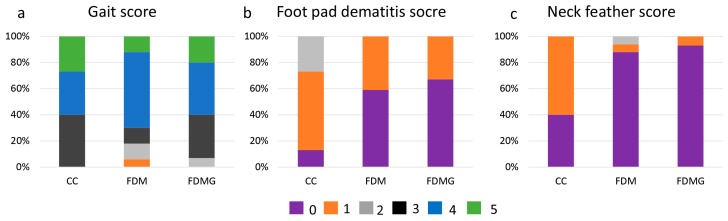
Gait score, foot pad dermatitis score, and feather condition score of chickens (*n* = 45). CC = conventional cage feeding, FDM = free dietary choice of mealworms feeding, FDMG = free dietary choice of mealworms and grass feeding and FRMG = free-range rearing system. Gait scores of 0, 1, 2, 3, 4, and 5 represent normal walking, abnormal walking, obviously lame, able to walk under strong stimulation, unable to walk, and unable to stand. Foot pad dermatitis scores of 0, 1, 2, 3, and 4 represent no injury, slight injury on <5% of the pads, a few injuries on 5–25% of the pads, moderate injuries on 25–50% of the pads, and severe injury on >50% of the pads. Feather scores of 0, 1, 2, 3, and 4 represent no feather loss, slight feather loss but not bare, size of bare patch < 3 × 3 cm, and size of bare patch > 3 × 3 cm.

Free dietary choice gave rise to significant differences in the gait score, i.e., FDM and FDMG chickens performed better than CC chickens (*p* < 0.01, Figure 1a). For foot pad dermatitis score, more than half of the hens in FDM (59%) and FDMG (67%) scored 0 (no injury), compared to only 13% in the CC group (Figure 1b). Neck feather condition scored better in FDM and FDMG hens than in CC hens (*p* < 0.05, Figure 1c). However, there was no significant difference between feathers from other parts among these groups.

#### 3.1.3. Physiological Characteristics

Free dietary choice affected physiology, with a significantly higher blood platelet value (×10^9^/L) in the examination of FDMG and FDM chickens, compared to CC chickens (*p* < 0.05) (Table 2).

#### 3.1.4. Gut Microbial Composition

**Figure 2:**
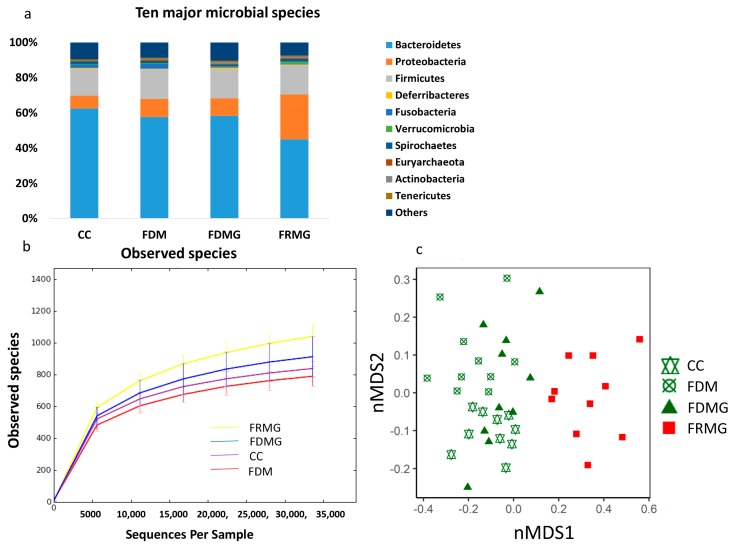
Gut microbial conditions of chickens (*n* = 10). (**a**) shows the 10 major microbial species in four rearing systems on phylum level; (**b**) shows the gut microbiome richness of each group by observed species of alpha-diversity (the richness of microbial composition: FRMG > FDMG > CC = FDM); (**c**) shows beta diversity by non-metric multidimensional scaling (NMDS) (Stress = 0.18).

Microbial analyses showed that *Bacteroidetes*, *Firmicutes*, and *Proteobacteria* were the dominant phyla (Figure 2a), and similar with class, order, family, genus, and species level. There was no significant difference in the 10 major microbial species among CC, FDM and FDMG groups. The gut microbiome was richer in FDMG than FDM and CC (*p* < 0.05) and did not differ between CC and FDM groups (Figure 2b). Beta diversity analyses revealed that chickens of CC and FDM had similar gut microbial structure within groups, yet different gut microbial structure between groups (Figure 2c). Chickens of FDMG had a relatively dispersive microbiome structure pattern within groups, of which some were similar to CC and some were similar to FDM (Figure 2c).

### 3.2. Cage and Free-Range Rearing

#### 3.2.1. Production Performance and Product Quality

Hens displayed lower FC and egg production and higher FC/EW in FRMG than FDMG (*p* < 0.05). The mortality and outcome of soft-shell eggs was much lower in FRMG than FDMG. A significantly higher average egg weight and egg yolk weight was observed in FRMG than FDMG (*p* < 0.05). In FRMG, egg yolk color was significantly lighter than FDMG at the age of 182 and 224 days (*p* < 0.05). In addition, greater cooking loss and lower shearing force was observed in chickens of FRMG than FDMG (*p* < 0.05), despite no difference in meat color (Table 3).

#### 3.2.2. Gait Score, Foot Pad Dermatitis Score, and Feather Condition Score

**Figure 3:**
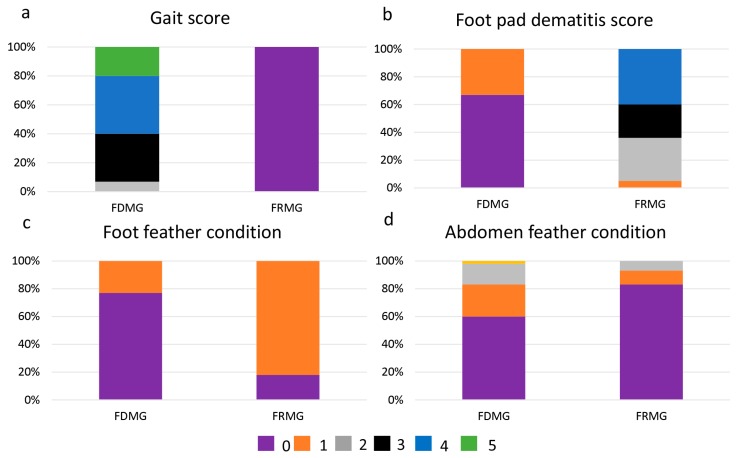
Gait score, foot pad dermatitis score, and feather condition score of chickens (*n* =45). FDMG = free dietary choice of mealworms and grass feeding and FRMG = free-range rearing system. Gait scores of 0, 1, 2, 3, 4, and 5 represent normal walking, abnormal walking, obviously lame, able to walk under strong stimulation, unable to walk, and unable to stand. Foot pad dermatitis scores of 0, 1, 2, 3, and 4 represent no injury, slight injury on <5% of the pads, a few injuries on 5–25% of the pads, moderate injuries on 25–50% of the pads, and severe injury on >50% of the pads. Feather scores of 0, 1, 2, 3, and 4 represent no feather loss, slight feather loss but not bare, size of bare patch < 3 × 3 cm, and size of bare patch > 3 × 3 cm.

Compared to FDMG, FRMG chickens had a significantly lower gait score (*p* < 0.01, Figure 3a). All chickens in FRMG scored 0, whereas no chicken in FDMG received this score (44% scored 3, 36% scored 4 and 15% scored 5). A large difference was displayed between FDMG and FRMG in relation to foot pad dermatitis score, with 46% of chickens scoring 0 in FDMG compared to 0% in FRMG (*p* < 0.05, Figure 3b). Nevertheless, 77% of FDMG chickens scored 0 on foot feather, which was significantly higher than FRMG chickens (18%, *p* < 0.05, Figure 3c). The abdomen feather score was significantly lower in FDMG with 60%, 15% and 2% scoring 0, 2 and 3, respectively, compared to 83%, 7% and 0% in FRMG chickens (*p* < 0.05, Figure 3d). However, there was no observed difference between other feather locations between groups.

#### 3.2.3. Physiological Characteristics

As compared to FDMG, FRMG chickens had a higher GRA (*p* < 0.05) and a lower MCH (%) (*p* < 0.05) and MCV (*p* < 0.05) values (Table 4).

#### 3.2.4. Gut Microbial Composition

The dominant microbial species within the free-range rearing system was the same as that of caged groups at phylum level (Figure 2a), and similar at the class, order, family, genus, and species levels. There was no significant difference in the 10 major microbial species between FDMG and FRMG groups. The alpha diversity was richer in FRMG than FDMG (*p* < 0.05). Moreover, beta diversity analyses revealed an apparent difference in gut microbial composition between free-range (FRMG) and cage-reared chickens (FDMG) (Figure 2c).

## 4. Discussion

In this study, our primary aim was to evaluate the effect of free dietary choice on welfare improvement under the cage rearing system. Further to this, we compared cage and free-range rearing systems under the same dietary choice, with the aim to provide a comprehensive understanding of welfare factors on the quantity and quality of chickens.

### 4.1. Free Dietary Choice under Cage Rearing Systems

Free dietary choice resulted in high feed conversion (FC/EW) compared to conventional cage feed rearing, while it was indicated that egg production and feed conversion were significantly better in two and three-choice diets, as compared to the single diet in laying hens [26]. However, both the FDMG and FDM hens contributed to lower mortality than CC hens, suggestive of benefits from free dietary choice on production efficiency. Free dietary choice had been reported to promote appetite, leading to foraging behaviors [27]. Even though these behaviors were not observed in this study, this may be the reason for the better gait score, foot pad condition, and neck and abdomen feather conditions in the two free dietary choice groups compared to the CC group. All the caged chickens suffered severe gait problems, while some of the free dietary chickens could still walk (score 1 and 2). This indicates that long-term cage rearing results in poor chicken welfare.

Although the microbial composition was poor in the caged chicken groups (compared to free-range chickens), free dietary choice (FDMG) chickens had a richer microbial composition than FDM and CC chickens. Gut health is known to influence the animal as a whole and alter its nutrient uptake and requirements, which is highly complex and encompasses the balance of the microbiome and the status of the immune system [28]. Specifically, commensal microbes are crucial for innate defenses and adaptive immune responses in chickens [29]. Therefore, the richer microbial composition in FDMG than in FDM and CC chickens implies a benefit of dietary choice in chickens. Platelets, which play a crucial role in physiological hemostasis [30], were higher in FDMG and FDM hens than in CC hens. Additionally, the higher plasma MCH and MCV, and lower GRA in FRMG than FDMG hens may be supported by a previous study in foxes, which indicated that stress resulted in the alteration of these physiological characteristics [31]. There is, however, scarce literature regarding the normal range of these parameters; therefore, we consider these blood indicators to be within the normal range, as all experimental chickens had no pathology.

Drip and cooking loss are important indicators of water-holding capacity in whole meat [32]. In the present study, FDMG chicken meat was revealed to have a higher water-holding capacity than FDM and CC chickens under the cage rearing system, suggestive of positive effects on meat quality based on dietary choice. The darker egg yolk color in FDMG than in FDG and CC chickens is likely to relate to the dietary choice of chicory, which was supported by a previous study indicating that mulberry leaves improved egg yolk color [33].

### 4.2. Cage and Free-Range Rearing

The lower feed conversion in FRMG than FDMG chickens was consistent with a previous study that revealed that free-range rearing resulted in a lower production performance and egg production rate than in cage-reared systems [34]. However, free-range hens also had heavier egg and yolk weight, lower mortality, and a lower number of total days producing soft-shell eggs, indicative of the benefits of the free-range system.

All the free-range chickens could walk normally, while caged chickens suffered from severe gait problems, which is evidence of the improvement of welfare under the free-range system. It has also been demonstrated that broiler chickens have a better gait score when they have an outdoor range [35]. That may be due to the limitations on natural behaviors under cage rearing versus the freedom to express natural behaviors under free-range rearing [36,37], even though such behaviors were not observed in this study. The poorer neck and abdomen feather condition in FRMG compared to FDMG chickens is probably for the same reason: a lack of access to sand leads to increased plumage deterioration [38]. The higher incidence of foot pad dermatitis found in FRMG compared to FDMG chickens was consistent with the finding that broiler chickens under free-range and organic systems showed a higher prevalence of foot pad dermatitis than those kept indoors [39]. This may be linked to the elevated temperature indoors caused by the fermentation bed designed to keep chickens warm during cold periods. In addition, inappropriate management, including heavy moisture of padding [40] and the opportunity to encounter extreme weather outdoors [11], could affect foot pad dermatitis in free-range chickens. In this study, a water tank was available in the outdoor area, thus chickens could freely drink, walk and play. Long-term contact between wet foot pads and soil may result in poorer foot pad condition, as seen from the poorer foot feather condition in FRMG than FDMG chickens.

The outdoor activities of free-range chickens are likely to be reflected in their health. Our study revealed an apparent difference in gut microbial composition between free-range and cage-reared chickens. This suggested an effect of rearing system on gut microbial composition. The microbial composition was richer in free-range chickens (FRMG) than in FDMG chickens, which is in accordance with a previous study indicative of heat stress in laying hens [41] as well as social stress in mice causing a significant change to their microbial composition [42] and a reduced alpha diversity of their gut microbiome [43]. This provided evidence that free-range rearing is conducive to a better balance of microbial composition, compared to stress-induced cage rearing [44].

Larger muscle fiber dimensions, relating to higher drip loss and cooking loss [32,45], were found in FRMG than in FDMG chickens. The higher red and lower shearing force values in meat from FRMG chickens were consistent with a previous study [46]. Thus, the above findings reveal that dietary choice and free-range rearing systems are conducive to good meat quality. Nevertheless, even though FDMG and FRMG chickens were fed the same diet, FRMG chickens had a lighter egg yolk color. Thus, a darker egg yolk color (which is favored by consumers) is influenced more by diet [33].

## 5. Conclusions

Under cage rearing, a free dietary choice of mealworms and fresh grass improved body condition, altered physiological characteristics, and enriched the gut microbial composition in chickens. These factors contributed to better meat quality. Under the same dietary program, the free-range rearing system was conducive to a better gait score, meat quality, and richer gut microbial composition. Therefore, we conclude that free dietary choice and free-range rearing systems improve the product quality, gait score, and microbial richness of chickens.

## Figures and Tables

**Table 1:** Production performance (*n* = 100), egg production and quality (*n* = 30) and meat quality (*n* = 10) among conventional cage feeding (CC), free dietary choice of mealworms feeding (FDM), and free dietary choice of mealworms and grass feeding (FDMG).

Items			CC	FDM	FDMG
Feed consumption (FC, g)			82.05 ^a^ ± 0.06	78.90 ^b^ ± 0.05	82.16 ^a^ ± 0.09
Egg weight (EW, g)			42.86 ^a^ ± 0.05	39.21 ^c^ ± 0.06	40.73 ^b^ ± 0.07
FC/EW			3.56 ^b^ ± 0.11	3.77 ^a^ ± 0.11	3.84 ^a^ ± 0.20
Egg production (%)			49.95 ± 1.00	51.07 ± 1.00	51.20 ± 1.01
Mortality (%)			18.09	9.57	12.77
Soft-shelled eggs (*n*)			186	193	211
Yolk weight (g)		Day 182 (Age)	10.88 ^a,b^ ± 0.07	10.42 ^b^ ± 0.09	11.27 ^a^ ± 0.07
		Day 224	12.47 ^a^ ± 0.88	12.15 ^a,b^ ± 1.13	11.79 ^b^ ± 1.06
		Day 266	14.03 ± 1.00	13.84 ± 0.09	13.54 ± 1.00
Yolk color		Day 182	7.29 ^b^ ± 0.05	9.27 ^a^ ± 0.07	9.54 ^a^ ± 0.09
		Day 224	6.20 ^b^ ± 0.09	6.20 ^b^ ± 1.01	7.75 ^a^ ± 0.08
		Day 266	6.24 ^b^ ± 1.00	7.08 ^b^ ± 0.06	7.79 ^a^ ± 0.07
Drip loss (%)		Thigh	2.29 ± 0.01	1.92 ± 0.01	2.11 ± 0.01
		Breast	2.54 ^a,b^ ± 0.02	2.22 ^b^ ± 0.02	2.91 ^a^ ± 0.01
Cooking loss (%)		Thigh	4.70 ± 0.03	4.42 ± 0.02	3.07 ± 0.03
		Breast	7.70 ± 0.02	6.48 ± 0.03	7.71 ± 0.03
Shearing force (kg/cm^2^)		Thigh	52.82 ± 0.05	49.82 ± 0.06	53.96 ± 0.05
		Breast	46.70 ± 0.05	44.55 ± 0.07	48.08 ± 0.05
Meat color	Light	Thigh	35.45 ^a^ ± 0.02	33.37 ^a,b^ ± 0.03	30.33 ^b^ ± 0.03
		Breast	42.86 ± 0.02	41.21 ± 0.02	40.26 ± 0.05
	Red	Thigh	3.63 ^b^ ± 0.21	4.28 ^b^ ± 0.20	5.07 ^a^ ± 0.12
		Breast	7.90 ± 0.34	7.44 ± 0.26	8.61 ± 0.33
	Yellow	Thigh	7.73 ± 0.05	7.43 ± 0.03	7.33 ± 0.03
		Breast	11.36 ^b^ ± 0.07	13.07 ^a^ ± 0.05	11.48 ^b^ ± 0.05

Different superscript letters a, b, c represents a statistically significant difference in the same line.

**Table 2:** Physiological characteristics between conventional cage feeding (CC), free dietary choice of mealworms (FDM), free dietary choice of mealworms and grass (FDMG) groups.

Items	CC	FDM	FDMG
LYM (%)	74.93 ± 1.18	74.18 ± 0.47	73.72 ± 0.51
MON (%)	5.62 ± 0.66	5.83 ± 0.12	5.72 ± 0.20
GRA (%)	19.38 ± 0.38	19.82 ± 0.48	20.13 ± 0.75
HGB (g/L)	144.55 ± 10.71	156.43 ± 5.40	149.10 ± 5.06
PLT (×10^9^/L)	169.40 ^b^ ± 48.81	261.00 ^a^ ± 17.61	217.00 ^a^ ± 28.71
MCH (pg)	58.25 ± 0.05	59.13 ± 0.05	58.54 ± 0.04
MCHC (g/L)	511.50 ± 6.34	504.29 ± 12.97	505.25 ± 6.80
RBC (×10^12^/L)	2.49 ± 0.01	2.58 ± 0.01	2.54 ± 0.02
MCV (FL)	114.13 ± 5.08	115.69 ± 4.33	116.08 ± 4.42

Different superscript letters a and b represents the statistical difference in the same line. LYM = lymphocyte, MON = monocyte, GRA = Granulocytes, HGB = hemoglobin, PLT = platelets, MCH = mean corpuscular hemoglobin, MCHC = mean corpuscular hemoglobin concentration, RBC = red blood cell count, and MCV = mean red cell volume. *n* = 10 in each group.

**Table 3:** Production performance (*n* = 100), egg production and quality (*n* = 30) and meat quality (*n* = 10) between cage rearing (FDMG) and free-range rearing system (FRMG).

Items			FDMG	FRMG
Feed consumption (FC, g)			82.16 ^a^ ± 0.09	75.10 ^b^ ± 0.07
Egg weight (EW, g)			40.73 ^b^ ± 0.07	45.49 ^a^ ± 0.07
FC/EW			3.84 ^b^ ± 0.20	4.63 ^a^ ± 0.30
Egg production (%)			51.20 ^a^ ± 1.01	37.45 ^b^ ± 1.02
Mortality (%)			12.77	5
Soft-shelled eggs (n)			211	4
Yolk weight (g)		Day 182 (Age)	11.27 ± 0.07	10.92 ± 0.07
		Day 224	11.79 ^b^ ± 1.06	12.59 ^a^ ± 1.12
		Day 266	13.54 ± 1.00	14.00 ± 0.08
Yolk color		Day 182	9.54 ^a^ ± 0.09	8.5 ^b^ ± 0.09
		Day 224	7.75 ^a^ ± 0.08	6.95 ^b^ ± 0.09
		Day 266	7.79 ± 0.07	8.07 ± 0.09
Drip loss (%)		Thigh	2.11 ± 0.01	2.03 ± 0.03
		Breast	2.91 ± 0.01	3.01 ± 0.02
Cooking loss (%)		Thigh	3.07 ^b^ ± 0.03	6.06 ^a^ ± 0.02
		Breast	7.71 ± 0.03	9.05 ± 0.03
Shearing force (kg/cm^2^)		Thigh	53.96 ^a^ ± 0.05	51.05 ^b^ ± 0.05
		Breast	48.08 ± 0.05	47.08 ± 0.05
Meat color	Light	Thigh	30.33 ± 0.03	33.28 ± 0.03
		Breast	40.26 ± 0.05	41.75 ± 0.04
	Red	Thigh	5.07 ^b^ ± 0.12	8.47 ^a^ ± 0.13
		Breast	5.80 ^b^ ± 0.33	8.61 ^a^ ± 0.34
	Yellow	Thigh	7.33 ± 0.03	8.00 ± 0.04
		Breast	11.48 ± 0.05	11.76 ± 0.07

Superscript letters a and b represent statistically significant differences.

**Table 4:** Physiological characteristics between cage rearing (FDMG) and free-range rearing system (FRMG).

Items	FDMG	FRMG
LYM (%)	73.72 ± 0.51	72.52 ± 1.06
MON (%)	5.72 ± 0.20	6.81 ± 0.68
GRA (%)	20.13 ^b^ ± 0.75	21.50 ^a^ ± 0.55
HGB (g/L)	149.10 ± 5.06	147.12 ± 5.35
PLT (×10^9^/L)	217.00 ± 28.71	267.33 ± 25.42
MCH (pg)	58.54 ^a^ ± 0.04	56.35 ^b^ ± 0.03
MCHC (g/L)	505.25 ± 6.80	513.00 ± 5.36
RBC (×10^12^/L)	2.54 ± 0.02	2.58 ± 0.01
MCV (FL)	116.08 ± 4.42	112.26 ± 3.46

Superscript letters a and b represent statistically significant differences in the same line. LYM = lymphocyte, MON = monocyte, GRA = Granulocytes, HGB = hemoglobin, PLT = platelets, MCH = mean corpuscular hemoglobin, MCHC = mean corpuscular hemoglobin concentration, RBC = red blood cell count, and MCV = mean red cell volume. *n* = 10 in each group.
